# High Carriage Rate of the Multiple Resistant Plasmids Harboring Quinolone Resistance Genes in *Enterobacter* spp. Isolated from Healthy Individuals

**DOI:** 10.3390/antibiotics11010015

**Published:** 2021-12-23

**Authors:** Yongyan Long, Xin Lu, Xiansheng Ni, Jiaqi Liu, Mengyu Wang, Xu Li, Zhe Li, Haijian Zhou, Zhenpeng Li, Kui Wu, Wei Wang, Liya Yang, Jialiang Xu, Haiying Chen, Biao Kan

**Affiliations:** 1 The Collaboration Unit for Field Epidemiology of State Key Laboratory for Infectious Disease Prevention and Control, Jiangxi Province Key Laboratory of Animal-Origin and Vector-Borne Disease, Nanchang Center for Disease Control and Prevention, Nanchang 330038, China; longyongyan@163.com (Y.L.); nccdcwjz@163.com (X.N.); mengyu_w0110@163.com (M.W.); wooky07@163.com (K.W.); music67@Sina.com (W.W.); 2 State Key Laboratory of Infectious Disease Prevention and Control, National Institute for Communicable Disease Control and Prevention, Chinese Center for Disease Control and Prevention, Beijing 102206, China; luxin@icdc.cn (X.L.); liujiaqi19990116@126.com (J.L.); lizhe900504@hotmail.com (Z.L.); zhouhaijian@icdc.cn (H.Z.); lizhenpeng@icdc.cn (Z.L.); yangliya29@126.com (L.Y.); 3 Beijing Technology and Business University, Beijing 102206, China; lixu@btbu.edu.cn (X.L.); xujialiang@btbu.edu.cn (J.X.); 4 School of Public Health, Shandong University, Jinan 250012, China

**Keywords:** PMQR genes, healthy participants, ciprofloxacin-resistant, *Enterobacter*

## Abstract

Antimicrobial-resistant bacteria causing intractable and even fatal infections are a major health concern. Resistant bacteria residing in the intestinal tract of healthy individuals present a silent threat because of frequent transmission via conjugation and transposition. Plasmids harboring quinolone resistance genes are increasingly detected in clinical isolates worldwide. Here, we investigated the molecular epidemiology of plasmid-mediated quinolone resistance (PMQR) in Gram-negative bacteria from healthy service trade workers. From 157 rectal swab samples, 125 ciprofloxacin-resistant strains, including 112 *Escherichia coli*, 10 *Klebsiella pneumoniae*, two *Proteus mirabilis*, and one *Citrobacter braakii*, were isolated. Multiplex PCR screening identified 39 strains harboring the PMQR genes (including 17 *qnr*,19 *aac(6′)-Ib-cr*, and 22 *oqxA*/*oqxB*). The genome and plasmid sequences of 39 and 31 strains, respectively, were obtained by short- and long-read sequencing. PMQR genes mainly resided in the IncFIB, IncFII, and IncR plasmids, and coexisted with 3–11 other resistance genes. The high PMQR gene carriage rate among Gram-negative bacteria isolated from healthy individuals suggests the high-frequency transmission of these genes via plasmids, along with other resistance genes. Thus, healthy individuals may spread antibiotic-resistant bacterial, highlighting the need for improved monitoring and control of the spread of antibiotic-resistant bacteria and genes in healthy individuals.

## 1. Introduction

Quinolones, a group of broad-spectrum antimicrobial agents, are widely used for treating infections caused by Gram-negative bacteria and are routinely used in animal breeding. In China, total antibiotic usage is estimated at approximately 162,000 tons/year [1], with human consumption accounting for approximately 48% and the remainder attributed to consumption by livestock and other domesticated animals [2]. Fluoroquinolone usage accounts for 17% of this total [3]. The acquisition of quinolone resistance may be caused by (i) chromosomal mutations in the bacterial genes encoding the protein targets of quinolones or by mutations that cause reduced drug accumulation via decreased uptake or increased efflux, or (ii) via the plasmid-located genes associated with quinolone resistance [4]. To date, three plasmid-mediated quinolone resistance (PMQR) mechanisms have been described: the first involves Qnr proteins, the second involves *aac(6′)-Ib-cr* genes, and the third involves *oqxA*, *oqxB*, and *qepA* plasmid-mediated efflux pumps [5,6]. Since the first PMQR gene was identified in a *Klebsiella pneumoniae* isolate [7], horizontal gene transfer has been considered the main route of quinolone resistance dissemination [8]. 

Numerous studies on the prevalence of PMQR genes in clinical settings and in animals have been reported worldwide [9,10,11,12]. However, few studies have investigated the prevalence of PMQR genes in healthy people. To ensure food safety, it is a regulatory requirement in China for healthy individuals in food-related industries to undergo health examinations to determine whether they harbor transferable drug resistance genes. In this study, we selected healthy individuals from the catering industry to investigate the prevalence of PMQR genes, and to estimate the potential threat that mobile quinolone resistance genes pose to the food industry.

## 2. Results

### 2.1. Bacterial Isolates 

In total, 125 non-duplicated ciprofloxacin (CIP)-resistant Gram-negative strains (including 112 *Escherichia coli*, 10 *K. pneumoniae*, two *Proteus mirabilis*, and one *Citrobacter braakii*) were isolated from 157 rectal swab samples; this corresponds to a carriage rate of 79.6% (125/157).

### 2.2. Antimicrobial Susceptibility Analysis

**Figure 1:**
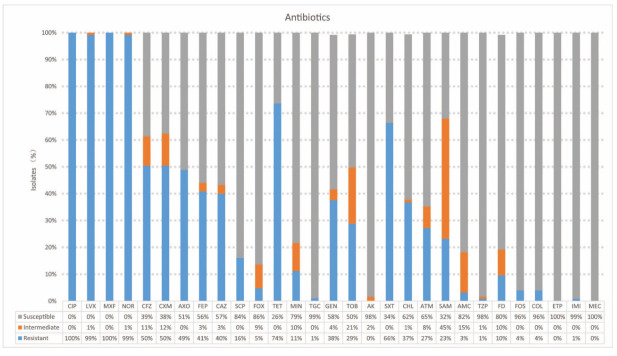
Antimicrobial-resistance phenotypes of the 125 bacterial isolates. Antimicrobial susceptibility testing was assessed using the following antimicrobial agents: amikacin (AK), amoxicillin-clavulanate (AMC), ampicillin-sulbactam (SAM), aztreonam (ATM), cefazolin (CFZ), cefepime (FEP), cefoperazone-sulbactam (SCP), cefoxitin (FOX), ceftazidime (FEP), ceftriaxone (AXO), cefuroxime (CXM), chloramphenicol (CHL), CIP, colistin (COL), ertapenem (ETP), fosfomycin with G6P (FOS), gentamicin (GEN), imipenem (IMI), levofloxacin (LVX), meropenem (MEC), minocycline (MIN), moxifloxacin (MXF), nitrofurantoin (FD), norfloxacin (NOR), piperacillin-tazobactam (TZP), tetracycline (TET), tigecycline (TGC), tobramycin (TOB), and trimethoprim-sulfamethoxazole (SXT).

All isolates were resistant to one or more quinolones or fluoroquinolones, including CIP (100%, 125/125), moxifloxacin (100%, 125/125), LVX (99.2%, 124/125), and levofloxacin (99.2%, 124/125), with the next highest resistance rates found for TET (73.6%, 92/125), SXT (66.4%, 84/125), GEN (37.6%, 47/125), and CHL (36.8%, 46/125). Some isolates exhibited resistance to cephalosporins, including cefazolin (50.4%, 63/125), FEP (40%, 50/125), and AXO (48.8%, 61/125). The antimicrobial susceptibility test results for the 29 tested antimicrobials are shown in Figure 1.

### 2.3. Prevalence of PMQR Genes

Among the 125 isolates, the overall detection rate for PMQR genes was 31.2% (39/125). PMQR genes were identified in 26 *E. coli* isolates (23.2%, 26/112), 10 *K. pneumoniae* (100%, 10/10), two *P. mirabilis* (100%, 2/2), and one *C. braakii* isolate (100%, 1/1) (Table 1).

The *qnr* subtypes detected included *qnrS1*, *qnrS2*, *qnrS13*, *qnrB6*, *qnrB52*, *qnrB91*, and *qnrA1*, with *qnrS1* predominating. None of the isolates were positive for *qnrC*, *qnrD*, or *qepA*. The *aac(6′)-Ib* gene was detected in 26 isolates. In most of these isolates (19 isolates, 73.1%), the mutated variant *aac(6′)-Ib-cr* of this gene was identified. There were 22 isolates that carried both *oqxA* and *oqxB* genes.

Overall, the most prevalent PMQR genes were *oqxB* (17.6%, 22/125), *oqxA* (17.6%, 22/125), *aac(6′)-Ib-cr* (15.2%, 19/125), and *qnr* (*qnrS1* = 7 *qnrS2* = 2, *qnrS13* = *1,qnrB6* = 4, *qnrB52* = 1, *qnrB91* = 1, and *qnrA1* = 1; 13.6%, 16/125), additionally, both *qnr2* and *qnr13* subtypes genes were detected in one *E.coli* strain. Among the 125 isolates, 26 (20.8%) carried two or more PMQR genes. Certain *E. coli* isolates (8.9%) carried only a single PMQR gene, whereas all *K. pneumoniae* isolates carried three or more PMQR genes (Table 1).

### 2.4. Other Acquired Resistance Genes Exist and Coexist in the Isolates

**Figure 2:**
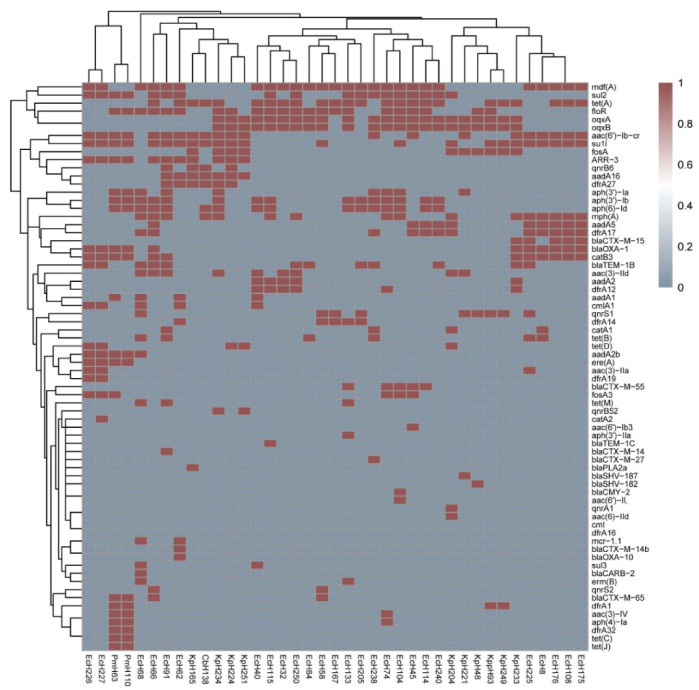
Heatmap showing the diversity profiles of drug resistance genes in the 39 PMQR gene-positive isolates.

We further identified coexisting antibiotic resistance genes based on the complete sequences of PMQR gene-harboring plasmids. Among all 39 PMQR gene-positive isolates (Table 2), the prevalence of beta-lactam genes in *E. coli* was 88.5% (23/26), with the main genotypes being *bla*_TEM-1B_ (40.7%), *bla*_OXA-1_ (29.6%), and *bla*_CTX-M-55_ (18.5%). One *K. pneumoniae* isolate was positive for *bla*_CTX-M-15_ + *bla*_OXA_ + *bla*_TEM-1B_, and three other isolates were positive for either *bla*_SHV182_, *bla*_SHV187_, or *bla*_PLA2a_. The sulfonamide gene detection rate in *K. pneumoniae* was 80% (8/10), with *sul*1 being the predominant genotype, whereas in *E. coli* it was 88.5% (23/26), with *sul*1 (44%) and *sul*2 (55.6%) predominating. The TET gene detection rate in *K. pneumoniae* was 80% (8/10), which included *tet(A)* (50%) and *tet(D)* (30%), whereas it was 88.5% (23/26) in *E. coli*, with *tet(A)* (66.7%) and *tet(B)* (22.2%) the main genotypes. While all *K. pneumoniae* isolates carried *fosA*, only five *E. coli* isolates carried *fosA3*. The trimethoprim gene detection rate in *K. pneumoniae* was 80% (8/10), with *dfrA1*, *dfrA12*, *dfrA17*, and *dfrA27* identified, whereas that for *E. coli* was 88.5% (23/26), with *dfrA17* (40.7%) the predominant genotype. The aminoglycoside gene detection rate in *K. pneumoniae* was 70% (7/10), with *aadA16* and *aac(3)-IId* the predominant genotypes, whereas that for *E. coli* was 88.5% (23/26), with *aph(3″)-Ib* combined with *aph(6)-Id* predominating. In addition, *floR* was the predominant phenicol-resistance gene in the *K. pneumoniae*, *E. coli*, and *P. mirabilis* isolates. The macrolide gene detection rate in *E. coli* was 100% (26/26), and *mph(A)*, *mdf(A)*, *erm(B)*, and *ere(A)* were detected. In addition, two *E. coli* isolates harbored the *mcr-1.1* gene. The distribution of drug resistance genes in the PMQR gene-positive isolates is shown in Figure 2. 

### 2.5. Drug Resistance Caused by Gene Mutations

Gene mutations in the DNA topoisomerase IV and gyrase encoding genes were found in all 26 *E. coli* strains (100%, 26/26). Ser at position 83 of the *E. coli* gyrase subunit A (*gyrA*) gene was mutated to Leu, and Asp at position 87 was mutated to Asn or Tyr. Ala at position 56 of the DNA topoisomerase IV, subunit A (*parC*) gene was mutated to Thr, Ser at position 80 was mutated to Ile, and Glu at position 84 was mutated to Val. Ser at position 458 of the topoisomerase IV, subunit B (*parE*) gene was mutated to Ala and Ile at position 529 was mutated to Leu. Among these, the *parC* and *gyrA* gene mutations were the most frequent.

### 2.6. Consistency Rates between the Drug Resistance Genes and Drug Resistance Phenotypes

By analyzing the correspondence between drug resistance genes and drug resistance phenotypes (Table 3), we found that the drug sensitivity results for 10 strains of *K. pneumoniae* to CIP, ertapenem, imipenem, meropenem, and GEN were completely consistent with the sequencing results (100%). The drug sensitivity test results for norfloxacin, however, were completely different from the sequencing results. The antibiotics whose drug sensitivity results were consistent with the sequencing results were cefoxitin (90%), amoxicillin-clavulanate (90%), piperacillin-tazobactam (90%), TET (70%), TGC (60%), CHL (90%), SXT (90%), FEP (50%), minocycline (50%), and amikacin (50%). The antimicrobial substances with a consistency rate of less than 50% were cefoperazone-sulbactam (40%), AXO (30%), aztreonam (10%), TGC (40%), and tobramycin (40%).

Additionally, the resistance patterns of the 26 *E. coli* strains to CIP, TGC, ertapenem, imipenem, meropenem, cefoxitin, TET, GEN, tobramycin, and myxin were completely consistent with the predicted results (100%). Over 50% of the drug sensitivity results from the other antibiotics were consistent with the predicted results, including cefepime (70.37%), FEP (66.67%), piperacillin-tazobactam (55.56%), CHL (77.78%), amikacin (55.56%), fosfomycin (92.59%), and SXT (88.89%).

## 3. Discussion

Antibiotic resistance is recognized as a ‘One Health’ challenge because of the rapid emergence and dissemination of resistant bacteria and genes that not only exists in humans (both in healthy individuals and patients), but also in wildlife, companion animals, agriculture (food-producing animals, fruits, and vegetables), and the environment (water and soil) on a global scale. *Enterobacter* spp. resistant to quinolones frequently arise in animals, being easily disseminated through the food-chain [13]. In rectal swab samples from some wildlife (non-human primates, mice) rectal swab samples, different variants of the *qnrB* gene were detected [14]. In the rectal swab samples selected from the companion animals, Gibson et al. reported that the *qnrA1* or *qnrB2* gene was often detected long with *aac(6′)-1b-cr* [15]. Approximately 4.1% (15/363) of strains isolated from the food-producing animals, such as swine, poultry, rabbit, and cattle, carried the *qnrB2*, *qnrB19*, and *qnrS1* genes, simultaneously [13]. In addition, fruits are usually consumed without cooking or processing, making them a potentially high-risk source in humans [16]. As mediators for the dissemination of antimicrobial-resistant *E. coli*, raw salads vegetable present a great risk to public health, and Nayme et al. detected PMQR determinants were detected in 52% of isolates [17]. In a study of wastewater samples from the swine feedlot, *qnrD*, *qepA*, *oqxB*, *qnrS,* and *oqxA* genes were detected [18]. Vaz-Moreira et al. found that the resistance strains could be disseminated from hospital effluent to aquatic environments, and that quinolone-resistant Gram-negative bacteria carried one or more of the PMQR genes [19]. Among 200 faecal samples randomly collected from chickens and pigs originating from different farms at the time of slaughter in Ibadan and Nigeria, PMQR genes were detected in 18 *E. coli* strains, which suggesting that the food animals may represent an important reservoir of PMQR in this region of Africa, and that antibiotic-resistant bacteria carried by animals can enter the human food chain through the consumption of meat or by direct contact [20]. PMQR genes (including *aac(6′)-1b-cr*, *qnrB,* and *qnrS*) were frequently detected in the herbs originating from Thailand (Water morning glory, Acacia and Betel leaf), Vietnam (Parsley, Asian pennywort, Houttuynia leaf, and Mint) and Malaysia (Holy basil and Parsley), suggesting that fresh culinary herbs from Southeast Asia are a potential source of contamination of food with quinolones resistant bacteria [21]. Because these herbs are generally consumed without appropriate heating, the resistant bacteria may be readily transferred to humans. Therefore, AMR Gram-negative bacteria can transfer from human-to-human directly or indirectly.

In this study, the isolation rate for Gram-negative strains with CIP resistance was alarmingly high at 79.6%. Almost all of the CIP-resistant strains were also resistant to moxifloxacin, norfloxacin, and levofloxacin. This may be related to the fact that these antibiotics are commonly used to treat gastroenteritis in adults [22]. Furthermore, low-dose antibiotics are added to animal feed as growth promoters and, while providing economic benefits, this accelerates the emergence of drug-resistant bacteria [13]. These factors contribute to the high prevalence of quinolone-resistant bacteria in healthy people. Multidrug resistance to different antibiotic categories was found in most isolates, particularly to commonly used antimicrobials such as TET, FEP, AXO, and cefepime. The minimum inhibitory concentration 50 (MIC_50_) and MIC_90_ for TET exceeded 16 µg/mL. Resistance to some commonly used clinical antibiotics, especially third- and fourth-generation cephalosporins, is concerning because this may lead to clinical treatment failure. To the best of our knowledge, few prevalence studies on PMQR genes in healthy people have been conducted. In this study, the PMQR gene detection rate was 31.2% and PMQR genes were identified in *E. coli*, *K. pneumoniae*, *P. mirabilis*, and *C. braakii* isolates. The PMQR gene carriage rate for *E. coli* in health practitioners was shown to be higher than that in healthy volunteers (14.3%) [23], and the reported detection rate for PMQR genes in animal breeders (55.9%) and the carriage rate in patients (51.4%) [24] were both higher than in our study [12]. The reason for these differences could be the high use of these antibiotics in clinical treatment and animal breeding. We found that the PMQR gene carriage rate for *K. pneumoniae* was higher than that for *E. coli*, which was consistent with previous studies showing carriage rates for *K. pneumoniae* and *E. coli* of 60.0–65.3% and 12.6–22.3%, respectively [25,26].

The most prevalent PMQR genes were *oqxB* and *oqxA*, followed by *aac(6′)-Ib-cr* and *qnr*. The detection rates for the *aac(6′)-Ib-cr* and *qnr* genes in *E. coli* were 9.8% and 6.25%, respectively, which were higher than the 9.0% and 0.85% rates recorded in Sweden and Norway [27]; these differences might be related to the scope and degree of antibiotic use in the different countries.

The prevalence rates for *aac(6′)-Ib-cr*, *oqxB*, and *oqxA* were slightly higher than those reported previously in healthy populations, but the prevalence of *qnr* was lower than that reported previously [28]. Some studies have shown that *oqxB* and *oqxA* are widely distributed among *E. coli* strains isolated from animal husbandry, with detection rates of 58.5% and 33.8%, respectively [29,30]. The *oqxB* and *oqxA* genes are also prevalent among *K. pneumoniae* isolates [31]. Of the 125 isolates we tested, two or more PMQR genes were present in 27 (21.5%) of the isolates. Some of the *E. coli* isolates (8.9%) only carried a single PMQR gene, whereas all of the *K. pneumoniae* isolates carried three or more PMQR genes, a result similar to previous findings [32].

According to the rules and regulations of the Public Places Sanitation Administration, the participants who work in the food processing and service trades must undertake a preventive health examination each year. In this study, we just focused on the individuals who work in the food processing and service trades; however, in future studies, we will expand our research to include all healthy individuals as the sample sources to represent the general healthy population.

In the present study, 39 PMQR-positive strains carried six to 16 types of acquired resistance genes, with most of the resistance genes co-existing on the same plasmid. The *tet(A)* and *bla_CTX-M-55_* genes were spread throughout the population, which may have public health significance. *K. pneumoniae* isolates also have a tendency to acquire resistance [33]. In this study, we found that the *sul1, floR*, and *tet(A)* genes were more common, which may also have public health significance. The most prevalent plasmid replicon types were IncFIB and IncFII in *E. coli* and IncFIB, IncR, and IncHI2 in *K. pneumoniae*. Other studies have also reported that these plasmids are the main types [31]. *aac(6′)-Ib-cr* and *qnr* were found to be chromosomally located in *E. coli*, which is in agreement with previous reports [34,35]. We found that *oqxA* and *oqxB* were chromosomally located in all strains except for one in which these genes were not detected. Previous studies have also reported that *oqxA* and *oqxB* are chromosomally located in *K. pneumoniae* [36]. In addition, two strains showed resistance to polymyxin, with *mcr*-*1.1* located on plasmids IncI2 and IncX4. Previous studies have reported that variant *mcr-1.6* was present in *Salmonella* isolated from healthy people [37]. Plasmids that carry *mcr-1.1* are a concern for healthy people because the frequent exchange of antibiotic resistance genes among *E. coli*, *K. pneumoniae*, and *Salmonella enterica* serovars could lead to the spread of polymyxin resistance [38].

## 4. Materials and Methods

### 4.1. Samples and Bacterial Strains

From May to August 2015, approximately 40 rectal swab samples were collected once a month; 157 rectal swab samples were collected at the Nanchang Center for Disease Control and Prevention (China) from healthy participants (55 males, 102 females; age range: 18–57 years), who received preventive health examination because of their occupation in food processing and other service trades, according to the rules of the administration for market regulation. None of the healthy individuals included in this study had been exposed to antibiotics or a hospital environment during the 3 months prior to sample collection. The swab samples were screened using ciprofloxacin (CIP) (4 µg/mL)-containing LB agar plates incubated at 37 °C for 18 h. For each sample, one transparent and smooth colony was selected, followed by identification using the API20E test (bioMerieux, France).

### 4.2. PMQR Gene Amplification

All of the selected isolates were screened for nine PMQR genes (*qnrA*, *qnrB*, *qnrC*, *qnrD*, *qnrS*, *aac(6′)-Ib*, *oqxA*, *oqxB*, and *qepA*) by PCR using the primers and reaction parameters reported in the references [9,28].

### 4.3. Antimicrobial Susceptibility Testing

For the PMQR gene-positive isolates, we conducted antimicrobial susceptibility testing with the following 29 antimicrobial agents: amikacin, amoxicillin-clavulanate, ampicillin-sulbactam, aztreonam, cefazolin, cefepime, cefoperazone-sulbactam, cefoxitin, ceftazidime (FEP), ceftriaxone (AXO), cefuroxime, chloramphenicol (CHL), CIP, colistin, ertapenem, fosfomycin with G6P, gentamicin (GEN), imipenem, levofloxacin, meropenem, minocycline, moxifloxacin, nitrofurantoin, norfloxacin, piperacillin-tazobactam, tetracycline (TET), tigecycline (TGC), tobramycin, and trimethoprim-sulfamethoxazole (SXT). We used the reference broth microdilution method with custom-made plates (BD, Phoenix NMIC-413). The breakpoints for each antimicrobial susceptibility test were selected based on the Clinical and Laboratory Standards Institute (CLSI, 2017 [39]) recommendations and the European Committee on Antimicrobial Susceptibility recommendations (EUCAST, 2018 [40]). *E. coli* ATCC25922 and *K. pneumoniae* ATCC700603 were used as quality control strains.

### 4.4. Genome and Plasmid Sequencing

DNA was extracted from 39 PMQR gene-positive isolates using the Wizard Genomic DNA Extraction Kit (Promega, Madison, WI, USA), followed by sequencing (HiSeq sequencer; Illumina, California, USA). Plasmid DNA was purified from 100 mL of liquid culture of the strain using the Qiafilter Plasmid max kit (Qiagen, Dusseldorf, German) as per the protocol for low-copy plasmids, and then sequenced using MinION (Oxford Nanopore, Oxford, UK) sequencers. MinION libraries of all plasmid DNAs were prepared using the SQK-LSK108 nanopore sequencing kit, R9 version (Oxford Nanopore, Oxford, UK). 1D base-calling was performed on a local computer in real-time by MinKNOW. We assembled each genome using a combination of short- and long-reads using the Unicycler hybrid assembler [41]. Plasmid replicons and drug resistance genes were identified in silico using online tools (http://www.genomicepidemiology.org/, accessed on 21 June 2021).

We sequenced the genomes of the PMQR gene-positive strains and the PMQR gene-harboring plasmids using both short- and long-read sequencing techniques. The complete sequences of all detected PMQR gene-positive plasmids have been deposited in the GenBank database, under BioSample numbers SAMN23848296, SAMN23848740, SAMN23848304, SAMN23848904, SAMN23848884, SAMN23895582, SAMN23895744, SAMN23895604, SAMN23895602, SAMN23895726, SAMN23895742, SAMN23895581, SAMN23895741, SAMN23895745, SAMN23895750, SAMN23895740, SAMN23895754, SAMN23895770, SAMN23895770, SAMN23895789, SAMN23895747, SAMN23895753, SAMN23895790, SAMN23895791, SAMN23895793, SAMN23895793, SAMN23895794, SAMN23895795, SAMN23895796, SAMN23895797, SAMN23895798, SAMN23895799, SAMN23895802, SAMN23895803, SAMN23895804, SAMN23895806, SAMN23895809, SAMN23895810, SAMN23847286.

## 5. Conclusions

In this study, we investigated the prevalence of quinolone-resistant, Gram-negative bacteria in healthy people. The prevalence rate of PMQR genes in healthy participants was relatively high, and the co-existence of resistance genes on plasmids is an important finding. Bacterial drug resistance in healthy people is a serious issue because it may unknowingly lead to the spread of antibiotic-resistant bacteria, which poses a serious public health threat. These findings highlight the necessity for improved monitoring of antibiotic-resistant bacteria in healthy individuals.

## Figures and Tables

**Table 1:** PMQR gene combinations in 125 bacterial isolates.

Bacterial Strains	No. of Isolates with Detected PMQR Gene ^a^							
	*qnr*	*aac*	*oqxA+* *oqxB*	*qnr+* *oqxA+* *oqxB*	*qnr+* *aac*	*oqxA+* *oqxB*+ *aac*	*aac*+ *qnr+* *oqxA+* *oqxB*	Total in Every Species, *n* (%)
*E. coli* (*n* = 112)	3 (2.7)	7 (6.3)	11 (9.8)	1 (0.9)	3 (2.7)	1 (0.89)	0 (0.0)	26 (23.2)
*K. pneumoniae* (*n* = 10)	0 (0.0)	0 (0.0)	1 (10)	4 (40)	1 (10)	1 (10)	3 (30)	10 (100)
*P. mirabilis* (*n* = 2)	0 (0.0)	2 (100)	0 (0.0)	0 (0.0)	0 (0.0)	0 (0.0)	0 (0.0)	2 (100)
*C. braakii* (*n* = 1)	0 (0.0)	0 (0.0)	0 (0.0)	0 (0.0)	1 (100)	0 (0.0)	0 (0.0)	1 (100)
Total (125)	3 (2.4)	9 (7.2)	12 (9.6)	5 (4.0)	5 (4.0)	2 (1.6)	3 (2.4)	39 (31.2)

^a^ aac, *aac(6′)-Ib-cr*.

**Table 2:** Characteristics of PMQR and other resistance genes in the plasmids of the *Enterobacteriaceae* isolates.

Strains	Incompatibility Group plasMINds and the Carrying Resistant Genes	Resistant Genes on Chromosome	Point Mutations of DNA Topoisomerase IV and Gyrase	MDR Phenotype
*K. pneumoniae*				
H93	IncR: ***qnrS****; sul1; tet(A); dfrA1* IncX1: *floR*	***OqxA*** *, **oqxB**; fosA*	-	CIP, LVX, NOR, MXF, TET, CHL, SXT
H165	IncN:***aac(6******′)-Ib-cr****, **qnrB6**; sul1; aadA16; tet(A); dfrA27; ARR-3* IncHI2A: -	*blaPLA2a; fosA*	-	CIP, LVX, NOR, MXF, TET, SXT
H204	IncHI1B:***qnrA**; aac(3)-IId, aadA5; catA1; mph(A); sul1, sul2, tet(D); dfrA17* IncHI2A: -	***OqxA*** *, **oqxB**; blaSHV-110, blaSHV-81;* *fosA*	-	CIP, GEN, LVX, MXF, NOR, MIN, CHL, FD, TET, SXT
H221	IncFIB: *aac(3)-IId, aph(3′)-Ia* IncHI2A: - IncFIB: -	***OqxA*** *, **oqxB**; blaSHV-187; fosA*	-	CIP, LVX, NOR, MXF, MIN, TET, CHL, GEN, FD, SXT
H224	IncR: ***aac(6′)-Ib-cr****, **qnrB6***; *aadA16; floR; sul1; tet(D); ARR-3; dfrA27*	***OqxA*** *, **oqxB**; fosA*	-	CIP, LVX, NOR, MXF, SAM, TET, MIN, CHL, SXT
H233	IncFIB: ***aac(6′)-Ib-cr****; blaCTX-M-15, blaOXA-1, blaTEM-1B; aadA2; catB3; mph(A); sul1; tet(A); dfrA12*	***OqxA*** *, **oqxB**; fosA*	-	CIP, NOR, MXF, LVX, SAM, ATM, CFZ, CAZ, AXO, CXM, TET, TOB, SXT
H234	IncR: ***aac(6′)-Ib-cr****, **qnrB52**; aac(3)-IId, aadA16, aph(3″)-Ib, aph(3′)-Ia, aph(6)-Id; floR; mph(A); sul; tet(A); dfrA27; ARR-3* IncFIB: -	***oqxA*** *, **oqxB**; fosA*	-	CIP, LVX, NOR, MXF, TET, CHL, GEN, FD, SXT
H249	IncR: ***qnrS****1; sul1; tet(A); dfrA1* IncHI2A: - IncL/M(pOXA-48): -	***oqxA*** *, **oqxB**; fosA*	-	CIP, LVX, MXF, NOR, FOX, CXM, TET, MIN, CHL, FD, SXT
H251	IncR: ***aac(6′)-Ib-cr****, **qnrB91**, aadA16; floR; sul1; tet(D); dfrA27; ARR-3* IncFIB: - IncHI1B: -	***oqxA*** *, **oqxB**; fosA*	-	CIP, LVX, NOR, MXF, MIN, TET, CHL, FD, SXT
*E. coli*				
H8	IncFII:***aac(6′)-Ib-cr**; blaOXA-1; aadA5; catA1, catB3; mph(A); sul1; tet(B); dfrA17* IncB: - IncO: - IncK: - IncZ: -	*mdf(A)*	*parC; gyrA; parE*	CIP, LVX, NOR, MXF, SAM, TET, TOB, SXT
H32	IncFIB: ***oqxA****, **oqxB**; aac(3)-IId, aadA2; floR; dfrA12; tet(A)* IncFII(29): -	*mdf(A)*	*gyrA; parC*	CHL, CIP, LVX, MXF, NOR, TET, GEN, TOB
H40	IncFIB: ***oqxA****, **oqxB**; aac(3)-IId, aadA, aadA2, aph(3″)-Ib, aph(6)-Id; cmlA1, floR; sul3; tet(A); dfrA12*	*mdf(A)*	*gyrA; parE; parC*	CHL, CIP, GEN, FD, MXF, LVX, NOR, TET, TOB, SXT
H45	IncFII: ***aac(6′)-Ib-cr****, **oqxA**, **oqxB**; blaCTX-M-55; aac(6′)-Ib3, aadA5; floR; sul2; tet(A); dfrA17; fosA3*	*mdf(A)*	*gyrA; parC*	CHL, CIP, MXF, LVX, NOR, ATM, CFZ, AXO, CXM, TET, TOB, FOS, SXT
H58	IncFIB: ***qnrS13****; dfrA14; tet(A); floR* IncHI2: ***oqxA****, **oqxB**, **qnrS2**; blaCTX-M-65; sul1; tet(A)*	*mdf(A)*	*gyrA; parC*	CIP, LVX, NOR, MXF, ATM, AXO, CFZ, CXM, TE, CHL
H62	p0111: *blaOXA-10; aadA1; tet(A); dfrA14; sul2; floR, cmlA1; ARR-2* IncI1: ***aac(6′)-Ib-cr****; blaCTX-M-14b; aadA16; sul; floR; dfrA27; ARR-3* IncI2: mcr-1.1 IncFIB: - IncFII: - Col(MG828): -	*mdf(A)*	*parE; parC; gyrA*	CIP, LVX, MXF, NOR, SAM, CFZ, FEP, AXO, CXM, TET, CHL, TOB, SXT, COL
H66	IncFIB: *blaTEM-1B*; *aadA5, aac(3)-IId, aph(3″)-Ib, aph(6)-Id; mph(A); sul2, sul1; tet(A)* IncHI1A: - IncHI1B: - IncX9: -	***aac(6′)-Ib-cr*** *, **qnrS2**; blaCTX-M-65, blaOXA-1; floR, catB3; mdf(A); ARR-3*	*parC; gyrA*	CIP, LVX, MXF, NOR, SAM, ATM, CFZ, AXO, CXM, TET, CHL, GEN, TOB, SXT
H68	IncN: *blaCARB-2; aac(3)-IId, aadA1, aadA2b, aph(3′)-Ia; sul3; tet(M); cmlA1, floR; dfrA17* IncFII: ***qnrS1****; blaTEM-1B; floR; mph(A), erm(B)* IncX1: *aph(3″)-Ib, aph(6)-Id; tet(B)* IncX4: *mcr-1.1*	*mdf(A)*	*parC; gyrA*	CIP, NOR, MXF, LVX, SAM, CHL, MIN, TET, GEN, TOB, SXT, COL
H74	IncFIB:***oqxA**, **oqxB**; aph(3′)-Ia, aph(4)-Ia, aac(3)-IV, aph(3″)-Ib, aph(3′)-Ia, aph(6)-Id; mph(A); sul2; tet(A); dfrA12; floR* IncFII: *blaCTX-M-55; fosA3*	*-*	*gyrA; parC*	CIP, LVX, NOR, MXF, SAM, ATM, FEP, CFZ, AXO, CXM, TET, CHL, GEN, TOB, FOS
H84	IncR: ***oqxA****, **oqxB**; blaTEM-1B; floR*	*mdf(A)*	*parC; gyrA*	CIP, LVX, NOR, MXF, TET, CHL
H91	IncFIA-IncHI1B-IncQ1: ***qnrB6****, **aac(6′)-Ib-cr**; aph(3′)-Ia, aac(3)-IId, aadA16, aph(3″)-Ib, aph(6)-Id; sul2; tet(B), tet(M); dfrA27; ARR-3* IncI1: *floR* IncX1: - Col440II: -	*blaTEM-1B; mdf(A), mph(A)*	*gyrA; parE; parC*	CIP, LVX, MXF, NOR, SAM, CFZ, SCP, AXO, CXM, MIN, TET, CHL, GEN, TOB, SXT
H104	IncA/C2: ***oqxA****, **oqxB**; blaCMY-2; sul1, sul2; aac(6′)-Il, aph(6)-Id, aph(3″)-Ib, aph(3′)-Ia; mph(A); tet(A)* IncFII/IncN: *blaCTX-M-55* Col156: -	*mdf(A)*	*gyrA; parC*	CIP, LVX, NOR, MXF, AMC, SAM, ATM, FEP, CFZ, FOX, SCP, CAZ, AXO, CXM, TET, CHL, TOB, FOS, SXT
H108	IncFIA: ***aac(6′)-Ib-cr****; blaOXA-1, blaCTX-M-15; aadA5; catB3; mph(A); sul1; tet(A); dfrA17* Col440II: -	*mdf(A)*	*parE; gyrA; parC*	CIP, LVX, NOR, MXF, FEP, SAM, ATM, CFZ, SCP, CAZ, AXO, CXM, TET, TOB, SXT
H114	IncFIB: ***oqxA****, **oqxB**; blaCTX-M-55; aadA5, aph(3″)-Ib, aph(6)-Id; sul2; tet(A); dfrA17; floR*	*mdf(A)*	*gyrA; parC*	CIP, LVX, NOR, MXF, ATM, CFZ, FEP, CAZ, AXO, CXM, TET, CHL, SXT
H115	IncN: ***oqxA****, **oqxB**; blaTEM-1C; aadA2, aph(3″)-Ib, aph(6)-Id; floR; mph(A); sul2; tet(A); dfrA12* Col440I: -	*mdf(A)*	*gyrA; parC*	CIP, LVX, NOR, MXF, TET, CHL, SXT
H133	IncFII-IncN: ***oqxA****, **oqxB**; blaCTX-M-55; aph(3″)-Ib, aph(3′)-IIa, aph(6)-Id; floR; sul2; tet(A); dfrA14* Col(MG828): -	*-*	*parC; gyrA*	CIP, LVX, NOR, MXF, ATM, CFZ, FEP, CAZ, AXO, CXM, TET, CHL, FD, SXT
H167	IncFIB: ***qnrS1****; floR; tet(A); dfrA14*	*mdf(A)*	*gyrA; parC*	CIP, LVX, NOR, MXF, TET, CHL
H175	IncFIA: *aadA5; mph(A); sul1; tet(A); dfrA17*	***aac(6′)-Ib-cr**; blaCTX-M-15, blaOXA-1; catB3; aac(3)-IIa; mdf(A)*	*parC; gyrA; parE*	CIP, LVX, NOR, MXF, ATM, CFZ, FEP, CAZ, AXO, CXM, TET, GEN, TOB, SXT
H176	IncFIA: *aadA5; mph(A); sul; tet(A); dfrA17* IncFII: -	***aac(6′)-Ib-cr**;blaCTX-M-15, blaOXA-1; catB3; aac(3)-IIa; mdf(A)*	*gyrA; parE; parC*	CIP, LVX, NOR, MXF, ATM, CFZ, FEP, CAZ, AXO, CXM, TET, GEN, TOB, SXT
H205	IncFIC: ***qnrS****; blaTEM-1B; aph(3″)-Ib, aph(6)-Id; sul; tet(A); dfrA14*	*mdf(A)*	*parE; gyrA; parC*	CIP, LVX, NOR, MXF, SAM, CFZ, FOX, AXO, CXM, TET, SXT
H225	IncFIA: ***aac(6′)-Ib-cr****; blaCTX-M-15, blaOXA-1; aadA5, aac(3)-IIa; catB3; sul; dfrA17; mph(A)* IncN:***qnrS1**; blaTEM-1B; mph(A)* IncL/M: -	*mdf(A); tet(B)*	*gyrA; parC; parE*	CIP, LVX, NOR, MXF, SAM, ATM, CFZ, FEP, CAZ, AXO, CXM, TET, GEN, TOB, SCP
H226	RepA: ***aac(6′)-Ib-cr****; blaOXA-1, blaTEM-1B; aac(3)-IIa, aadA2b; catA2, catB3, cmlA; ere(A); sul1, sul2; tet(D); dfrA19; fosA3; ARR-3* IncX1: -	*mdf(A)*	*gyrA; parC*	CIP, LVX, NOR, MXF, SAM, TET, CHL, GEN, TOB, SXT
H227	RepA: ***aac(6′)-Ib-cr****; blaOXA-1, blaTEM-1B; aac(3)-IIa, aadA2b; catA2, catB3, cmlA; ere(A); tet(D); sul1, sul2; dfrA19; fosA3; ARR-3* IncX1: -	*mdf(A)*	*gyrA; parC*	CIP, LVX, NOR, MXF, SAM, TET, CHL, GEN, TOB, SXT
H238	IncQ1:***oqxA**, **oqxB**; blaTEM-1B, blaCTX-M-27; aph(3″)-Ib, aph(3′)-Ia, aph(6)-Id; catA1; sul2; tet(B); dfrA17* IncFIB: -	*-*	*gyrA; parC*	CIP, LVX, NOR, MXF, ATM, CFZ, FEP, AXO, CXM, CHL, TET, SXT
H240	IncQ1: -	***oqxA*** *, **oqxB**; blaTEM-1B; aadA5, aph(3″)-Ib, aph(6)-Id; tet(A); mdf(A); sul2; dfrA17*	*gyrA; parC*	CIP, LVX, NOR, MXF, SAM, TET, FD, SXT
H250	IncFIB: ***oqxA****, **oqxB**; blaTEM-1B; aac(3)-IId, aadA2; mph(A), tet(A); dfrA12* IncI1: *floR; sul2*	*mdf(A)*	*parC; gyrA; parE*	CIP, LVX, NOR, MXF, NOR, CHL, TET, GE, TOB, FD, SXT
*C. braakii*				
H138	**IncR**:***qnrB6**, **aac(6′)-Ib-cr**; aadA16, aph(6)-Id; mph(A); sul1; tet(A); dfrA27; ARR-3; floR*	*-*	*-*	CIP, LVX, MFX, NOR, AMC, CFZ, FOX, TET, CHL, SXT

MDR: multidrug resistance. This is defined in a strain as resistance to more than three different antibiotics. Antimicrobial susceptibility testing was assessed in the PMQR isolates using the following antimicrobial agents: amikacin (AK), amoxicillin-clavulanate (AMC), ampicillin-sulbactam (SAM), aztreonam (ATM), cefazolin (CFZ), cefepime (FEP), cefoperazone-sulbactam (SCP), cefoxitin (FOX), ceftazidime (FEP), ceftriaxone (AXO), cefuroxime (CXM), chloramphenicol (CHL), CIP, colistin (COL), ertapenem (ETP), fosfomycin with G6P (FOS), gentamicin (GEN), imipenem (IMI), levofloxacin (LVX), meropenem (MEC), minocycline (MIN), moxifloxacin (MXF), nitrofurantoin (FD), norfloxacin (NOR), piperacillin-tazobactam (TZP), tetracycline (TET), tigecycline (TGC), tobramycin (TOB), and trimethoprim-sulfamethoxazole (SXT). The Plasmidfinder detection results (Table 2) showed that, apart from two *P. mirabilis* strains, one to six plasmid replicon types were present in all strains. In all 39 PMQR gene-positive isolates, the highest detection rate was for IncFIB (*n* = 15), followed by IncFII (*n* = 9), IncR (*n* = 7), IncN (*n* = 6), and IncFIA (*n* = 5). We sequenced all 39 PMQR gene-positive strains, yielding 31 complete sequences and two draft sequences of the PMQR gene plasmids, additionally, two *E. coli* strains possessed two PMQR gene plasmids. The PMQR genes were located on the chromosome in four *E. coli* strains, two *P. mirabilis* strains, and one *K. pneumoniae* strain. The location of the PMQR gene could not be determined in one *K. pneumoniae* strain. The PMQR genes were detected both on plasmids and the chromosome in seven *K. pneumoniae* strains. The complete sequences of seven IncFIB, six IncR, three IncN, three IncFII, two IncFIA, two RepA, one IncQ1, one IncFIA-IncHI1B-IncQ, one IncFII- IncN, one IncHI1B, one IncHI2, one IncFIC, and one IncI1 PMQR-harboring plasmid were obtained, with lengths of 59,494–224,070, 53,007–96,613, 49,618–168,792, 25,700–141,489, 123,952–167,131, 250,898, 161,298, 290,674, 126,885, 285,541, 233,321, 118,361, and 115,027 bp, respectively. Seven IncFIB PMQR plasmids carried 5–12 resistance genes, six IncR PMQR plasmids carried 4–13 resistance genes, three IncN PMQR plasmids carried 2–11 resistance genes, three IncFII PMQR plasmids carried 4–11 resistance genes, two IncFIA PMQR plasmids carried nine resistance genes, two RepA PMQR plasmids carried 15 resistance genes, one IncQ1 PMQR plasmid carried 11 resistance genes, one IncFIA-IncHI1B-IncQ1 PMQR plasmid carried 12 resistance genes, one IncFII-IncN PMQR plasmid carried 10 resistance genes, one IncHI1B PMQR plasmid carried 10 resistance genes, one IncHI2 PMQR plasmid carried six resistance genes, one IncFIC PMQR plasmid carried four resistance genes, and one IncI1 PMQR plasmid carried six resistance genes. The highest detection rates in *K. pneumoniae* were for IncFIB (50%, 5/10) and IncR (50%, 5/10). The *aac(6′)-Ib-cr* and *qnr* genes were mostly located on the IncFIB plasmid (5/10, 50%). All *oqxA* and *oqxB* genes were located on the *K. pneumoniae* chromosome (except for *K. pneumoniae* H48 where the location was not defined). In five *K. pneumoniae* isolates, the *aac(6′)-Ib-cr* and *qnr* genes coexisted with *aadA16*, *dfrA1*, *dfrA27*, *sul1*, *tet(A)*, *tet(D)*, *floR*, and *ARR*-3 resistance genes on the IncR plasmid. In *E. coli*, IncFIB (10/26, 37.0%) and IncFII (9/26, 34.6%) were the main replicon types, and the PMQR genes were mainly located on IncFIB (7/26, 26.9%) and IncFII (5/26, 19.2%). In seven *E. coli* isolates, *oqxA*, *oqxB*, and *qnr* coexisted with *aadA2*, *aac(3)-IId*, *aph(6)-Id*, *aph(3″)-Ib*, *catA1*, *catB3*, *floR*, *sul2*, *tet(A)*, *mph(A)*, *dfrA12*, *dfrA17*, and *floR* resistance genes on the IncFIB plasmid. In five *E. coli* isolates, *oqxA*, *oqxB*, *aac(6′)-Ib-cr*, and *qnr* coexisted with *bla*_CTX-M-55_, *bla*_TEM-1B_, *aadA5*, *aph(6)-Id*, *aph(3″)-Ib*, *sul2*, *dfrA14*, *dfrA17*, *tet(A)*, *mph(A)*, and *floR* resistance genes on the IncFII plasmid. In three *E. coli* isolates, *aac(6′)-Ib-cr* and *qnr* coexisted with *bla*_OXA-1_, *aadA5*, *sul1*, *dfrA17*, *tet(A)*, *catB*3, and *mph(A)* resistance genes on the IncFIA plasmid.

**Table 3:** Concordance rate between drug resistance genes and drug resistance phenotypes.

Drug Resistance ^a^	*K. pneumoniae*	*E. coli*
CIP	100%	100%
NOR	0%	\
FEP	40%	69.20%
FOX	90%	100.00%
CAZ	50%	65.40%
AXO	30%	\
AMC	90%	\
TZP	90%	53.80%
ATM	50%	\
TET	70%	100%
TGC	60%	100%
MIN	30%	\
CHL	90%	76.90%
AK	50%	53.80%
GEN	100%	100%
TOB	40%	100%
ETP	100%	100%
IMI	100%	100%
MEC	100%	100%
FOS	10%	92.30%
SXT	90%	88.40%
COL	\	100%

^a^ Antimicrobial susceptibility testing was assessed using the following antimicrobial agents: amikacin (AK), amoxicillin-clavulanate (AMC), ampicillin-sulbactam (SAM), aztreonam (ATM), cefazolin (CFZ), cefepime (FEP), cefoperazone-sulbactam (SCP), cefoxitin (FOX), ceftazidime (FEP), ceftriaxone (AXO), cefuroxime (CXM), chloramphenicol (CHL), CIP, colistin (COL), ertapenem (ETP), fosfomycin with G6P (FOS), gentamicin (GEN), imipenem (IMI), levofloxacin (LVX), meropenem (MEC), minocycline (MIN), moxifloxacin (MXF), nitrofurantoin (FD), norfloxacin (NOR), piperacillin-tazobactam (TZP), tetracycline (TET), tigecycline (TGC), tobramycin (TOB), and trimethoprim-sulfamethoxazole (SXT).

## Data Availability

The datasets used and/or analyzed during the current study available from the corresponding author on reasonable request.
